# Body Mass Index and Up-to-Date Colorectal Cancer Screening Among Marylanders Aged 50 Years and Older

**Published:** 2006-06-15

**Authors:** Eileen K Steinberger, Mikhail Menis, Bernard Kozlovsky, Pat Langenberg, Min Zhan, Ebenezer Israel, Annette Hopkins, Diane M Dwyer, Carmela Groves

**Affiliations:** Department of Epidemiology and Preventive Medicine, University of Maryland School of Medicine, Howard Hall; America’s Health Insurance Plans, Washington, DC; Department of Epidemiology and Preventive Medicine, University of Maryland School of Medicine, Baltimore, Md; Department of Epidemiology and Preventive Medicine, University of Maryland School of Medicine, Baltimore, Md; Department of Epidemiology and Preventive Medicine, University of Maryland School of Medicine, Baltimore, Md; Department of Epidemiology and Preventive Medicine, University of Maryland School of Medicine, Baltimore, Md; Department of Epidemiology and Preventive Medicine, University of Maryland School of Medicine, Baltimore, Md; Center for Cancer Surveillance and Control, Maryland Department of Health and Mental Hygiene, Baltimore, Md; Center for Cancer Surveillance and Control, Maryland Department of Health and Mental Hygiene, Baltimore, Md

## Abstract

**Introduction:**

Overweight and obese individuals are at increased risk for developing and dying from colorectal cancer. Studies suggest that overweight and obese women are more likely to avoid or delay cancer screening. Our objective was to determine whether overweight or obese adults aged 50 years and older living in Maryland in 2002 were less likely to be up-to-date with colorectal cancer screening than normal and underweight adults.

**Methods:**

The relationship between body mass index and colorectal cancer screening was evaluated based on responses from 3436 participants aged 50 years and older to the Maryland Cancer Survey 2002, a population-based random-digit–dial telephone survey. The survey contains self-reported information on colorectal cancer screening, height, weight, and potential confounders. Logistic regression was performed to calculate odds ratios (ORs) and 95% confidence intervals (CIs), adjusted for age, sex, race, employment, marital status, education, area of residence, and health-care–related variables.

**Results:**

Overall, 64.9% of Marylanders aged 50 and older were up-to-date with colorectal cancer screening. Compared with normal and underweight individuals, overweight individuals had similar odds of being up-to-date with colorectal cancer screening (OR, 1.05; 95% CI, 0.83–1.33). Obese individuals had slightly lower odds, but this difference was not statistically significant (OR, 0.84; 95% CI, 0.65–1.09). Recommendation by a health care provider for colorectal cancer screening was strongly associated with up-to-date colorectal cancer screening (OR, 36.7; 95% CI, 28.7–47.0).

**Conclusion:**

Our study shows no statistically significant association between body mass index levels and up-to-date colorectal cancer screening. We recommend that physicians and other health care providers increase up-to-date colorectal cancer screening rates in the population by referring their patients for appropriate screening.

## Introduction

Colorectal cancer (CRC) is the third most common type of cancer among men and among women and is the second leading cause of cancer-related mortality in the United States (1,2). It has been estimated that in 2004, about 146,940 new cases were diagnosed, and about 56,730 individuals died of CRC (2). Approximately 93% of CRC cases occur in people who are aged 50 years or older (3). Therefore, asymptomatic individuals without known risk factors (e.g., a personal or family history of CRC or adenomas, certain genetic syndromes, personal history of inflammatory bowel disease, endometrial or ovarian cancer) are recommended to undergo CRC screening beginning at age 50 (4,5). This recommendation includes either a fecal occult blood test (FOBT) annually, a flexible sigmoidoscopy every 5 years, an annual FOBT plus flexible sigmoidoscopy every 5 years, a colonoscopy every 10 years, or a double-contrast barium enema (DCBE) every 5 years, as recommended by the American Cancer Society. CRC screening has been shown to reduce CRC mortality through identifying and removing precancerous polyps and detecting and treating the cancer in its early stages (4,5).

The prevalence of obesity in the United States has increased more than 30% in the past decade (6). An estimated 64% of U.S. adults aged 20 and older were overweight or obese in 1999 to 2000 (7). Overweight and obese individuals with a body mass index (BMI) of 25 kg/m^2^ or higher are at increased risk for some types of cancers including endometrial, breast, prostate, gallbladder, and colorectal (8,9). Studies suggest that higher body weight is associated with higher mortality from CRC (10-13). Because overweight and obese individuals are at higher risk for developing CRC as well as at higher risk of dying from CRC, it is important that they obtain the recommended CRC screening.

Previous studies suggested that overweight and obese women are more likely to avoid or delay cancer screening (14-17). However, only one of these studies specifically evaluated the relationship between BMI and CRC screening; this study showed that morbidly obese women were less likely to be screened for CRC (17). The primary purpose of our study was, therefore, to determine whether overweight or obese adults aged 50 and older living in Maryland in 2002 were less likely to have an up-to-date CRC screening than normal and underweight adults. We used the Maryland Cancer Survey (MCS) 2002 as a primary data source to evaluate the relationship between BMI and CRC screening.

## Methods

### MCS survey design and data collection 

The MCS 2002 (18) is a population-based statewide survey on cancer screening and behavioral risk factors among people aged 40 and older. Methods for the survey are based on the Behavioral Risk Factor Surveillance System (BRFSS). Institutional review board approval was received from the University of Maryland and the Maryland Department of Health and Mental Hygiene.

The MCS was conducted by telephone using random-digit–dialing with computer-assisted telephone interview (CATI) and list-assisted disproportionate stratified sampling. Respondents were eligible to participate in the survey if they were aged 40 or older and lived in a private residence in Maryland. Respondents were ineligible for participation if they were younger than 40 years, did not speak English, lived in group homes and institutions, or were unable to communicate because of a physical or mental impairment. 

For the purposes of sampling, Maryland was divided into two geographic strata: urban and rural. The urban area consisted of Baltimore City and seven counties in the Metropolitan Baltimore–Washington, D.C., area (Anne Arundel, Baltimore, Carroll, Harford, Howard, Montgomery, and Prince George's). The rural area consisted of the remaining counties in western Maryland, southern Maryland, and the Eastern Shore. The Marketing Systems Group, Genesys Sampling (Fort Washington, Pa), provided 100,000 random telephone numbers. Each geographic stratum had three types of telephone number *blocks*: *listed one plus*, *not listed one plus*, and *zero*. These blocks corresponded to the number of residential telephone numbers found among a series of 100 numbers. *One plus* blocks were known to contain at least one residential telephone number and were sampled at a higher rate than the *zero* block telephone numbers. Rural telephone numbers were oversampled.

Telephone interviews using CATI were conducted by REDA International, Inc (Wheaton, Md), a survey research firm. When someone answered the telephone, the number was confirmed to be a residential phone number. It was determined whether an adult aged 40 years or older was living in the residence. If two or more people in the household were aged 40 or older, one was selected at random for the anonymous interview, which took about 20 minutes to complete. A total of 84,172 numbers were called or prescreened as nonresidential numbers: 51.8% were nonworking numbers, 10.3% were business or institution phone numbers, and 26.3% of were ineligible or excluded for various reasons. Overall, 6.0% of phone numbers resulted in 5071 completed interviews for the survey. Responses from 31 people who refused to give their race were omitted from the data set for analysis, leaving 5040 respondents. The Council of American Survey Research Organizations (CASRO) response rate, defined as Completed Interviews/(Eligible + Presumed Eligible), was 38.4%. The completion rate, defined as Completed Interviews/Known Eligible, was 65.4%.

The data set was weighted according to BRFSS weighting protocol (19). The final weight was applied to take into account the sampling probability by geographic region (urban or rural), residential telephone sampling among the three blocks of phone numbers (listed one plus, not listed one plus, and zero block), the number of adults aged 40 and older in the respondent's household, the number of residential telephone numbers in each household, and the number of people in age, race, and sex categories for each geographic stratum.

### Statistical analysis 

**Definition of the analytic sample**

A total of 5040 respondents aged 40 years and older participated in the MCS 2002. This secondary data analysis was performed on 3436 people whose age in the data set was 50 years or older, the age at which CRC screening is recommended.

**Definition of the outcome variable**

Respondents were asked about testing with FOBT and sigmoidoscopy or colonoscopy and the time since their most recent test. Individuals were considered to have up-to-date colorectal cancer screening if they had received an FOBT within the last year, a sigmoidoscopy within the last 5 years, or a colonoscopy within the last 10 years. Individuals who answered "don't know/not sure" or "refused" to the questions about specific cancer screening tests or were unable to specify the time interval since their last screening examination (answered "don't know/not sure" or "refused") were excluded from the up-to-date screening analysis (n = 100).

**Definitions of the main study variables**

Definitions of overweight and obesity are based on the commonly used BMI. BMI is equal to weight in kilograms divided by height in meters^2^. BMI was subdivided into three weight categories: normal weight or underweight (BMI <25), overweight (BMI 25–29.9), and obese (BMI ≥30) (20).

**Potentially confounding or effect-modifying variables**

We first identified potentially confounding or effect-modifying covariates from the MCS data based on the scientific literature (21-23), our views of their importance, and their availability in the MCS. The covariates identified were geographic area of residence (urban or rural), sex, race, age, marital status, education, employment, general health status, health insurance, physical examination in the last 2 years, and CRC screening recommendation by a physician or other health professional. Respondents were considered to have had a recommendation for CRC screening if they had ever received a recommendation for a sigmoidoscopy or colonoscopy or if they had received a recommendation for an FOBT in the last year. We then ascertained the unadjusted associations of these potential confounding or effect-modifying variables with up-to-date CRC screening and BMI levels. Variables found to be significantly associated (*P* < .05) with up-to-date CRC screening, as well as sex and geographic area, were included in the final multivariable analysis. We also examined whether any variables modified the association between BMI and CRC screening by conducting stratified analyses. In the stratified analyses, only sex was found to modify this association.

**Analysis**

The bivariate analyses with weighted proportions were assessed for significance using the chi-square test and analyzed with SAS version 9.1 (SAS Institute, Cary, NC). The association between BMI levels and up-to-date CRC screening was estimated using multivariable logistic regression models to control for potentially confounding factors. The final model controlled for sex, race, age, marital status, education, employment, geographic area, health insurance, having had a physical examination in the last 2 years, and CRC screening recommendation. The interaction terms between sex and BMI were excluded from the final models because the overall modifying effect by sex was not significant after controlling for all other covariates in the logistic regression model. The unweighted multivariable analyses were performed using SAS version 9.1.

## Results

There were 3436 Marylanders aged 50 years and older who participated in the MCS 2002. The surveyed sample is described in Table 1; 78.9% were white, 62.4% were women, 56.3% were aged 50 to 64, and 56.8% were married or a partner in an unmarried couple. The weighted percentages in Table 1 show that about 24.0% of Marylanders aged 50 and older belonged to the highest income category of $75,000 or more; 45.2% were retired, 43.0% were employed, 57.3% had some college education or more, and 77.9% lived in urban areas.

Table 2 displays the health-care–related characteristics of Marylanders aged 50 and older. Among this population, 95.8% reported having some type of health insurance; 86.8% had visited a health care provider in the last year for a routine checkup, and 93.7% reported having had a routine checkup in the last 2 years. In addition, 40.9% reported having received a recommendation in the last year to perform a home FOBT, and 63.5% reported that at some time they had received a recommendation to have a lower gastrointestinal (GI) endoscopy (sigmoidoscopy or colonoscopy). Combining these two recommendations, 73.2% reported either receiving a recommendation in the last year to have an FOBT or ever receiving a recommendation to have a lower GI endoscopy.

About 33.8% of Marylanders aged 50 and older had received an FOBT within the preceding year; 11.4% had received a sigmoidoscopy within the last 5 years; and 42.0% had received a colonoscopy within the last 10 years (data not shown). Overall, 64.9% of Marylanders aged 50 and older had received an up-to-date CRC screening with FOBT, sigmoidoscopy, or colonoscopy. The remainder either reported never having had CRC screening (25.9%) or having been tested in the past but not being up-to-date (9.3%). Race, age, marital status, education, employment status, health insurance, physical examination in the last 2 years, and receiving a screening recommendation from a health care provider were significantly associated with up-to-date CRC screening (Table 3). People of other races were less likely than whites or blacks to have had an up-to-date screening. Adults who were aged 65 and older, were married, were currently retired, had higher levels of education, or had health insurance were more likely to have had an up-to-date CRC screening. Up-to-date CRC screening was not associated with area of residence, sex, or self-reported health status. Individuals who had received a physical examination in the last 2 years were more likely to have received an up-to-date CRC screening than individuals who did not have a physical examination in the last 2 years, and individuals who reported receiving a health care provider screening recommendation for either FOBT or lower GI endoscopy were more likely to have received an up-to-date CRC screening than those who reported no provider recommendation. The association with the health care provider recommendation was the strongest.

More than 60% of Marylanders aged 50 and older were either overweight (36.0%) or obese (25.4%) (Table 4). Fewer than 40% of Marylanders aged 50 and older were either of normal weight (35.3%) or underweight (3.3%). Sex, race, age, marital status, education, and health status were significantly associated with BMI levels. Men were more likely to be overweight, whereas women were more likely to be normal or underweight; blacks were more likely to be obese than whites or people of other races; individuals with education beyond high school tended to be normal or underweight more often than those with a high school education or less; and individuals in excellent or very good or good health status tended to be normal or underweight compared with individuals who considered themselves in fair or poor health status, who were more likely to be obese. No significant differences in BMI distribution were found by area of residence, employment status, health insurance, time since last physical examination, or screening recommendation from a provider.

Table 5 displays the unadjusted associations between BMI levels and CRC screening. There were no significant differences in up-to-date CRC screening rates among different BMI categories (*P* = .84). Table 6 shows the results of two multivariable logistic regression models examining the association between BMI levels and up-to-date CRC screening adjusted for potentially confounding factors. The first model (model 1) uses only sociodemographic characteristics, whereas the second includes health-care–related variables (i.e., health insurance, physical examination in the last 2 years, and provider recommendation for CRC screening). In the first model, overweight and obese individuals had similar odds of having an up-to-date CRC screening as normal or underweight individuals (OR, 1.07; 95% CI, 0.89–1.27; and OR, 1.07; 95% CI, 0.88–1.30, respectively). In the second model (model 2), the odds of having up-to-date screening for overweight individuals compared with normal and underweight individuals were similar. Obese individuals had slightly lower odds of having up-to-date screening than normal and underweight individuals (OR, 0.84; 95% CI, 0.65–1.09), but this difference was not statistically significant (*P* = .19).

The addition of health-care–related variables in model 2 does not significantly alter the association between BMI levels and up-to-date CRC screening. Higher odds of having up-to-date CRC screening were found when a routine physical examination had been received in the last 2 years (OR, 2.53; 95% CI, 1.65–3.90). Reporting a recommendation for CRC screening by a health care provider resulted in the highest OR of 36.7 (95% CI, 28.7–47.0).

The results of both logistic regression analyses show that adults aged 65 and older had higher odds of having an up-to-date CRC screening than individuals aged 50 to 64. Higher odds of having an up-to-date CRC screening were also found among individuals with at least some college education than among individuals with a high school education or less. Lower odds of having an up-to-date CRC screening were found among individuals who were employed for wages, self-employed, and not employed than among individuals who were retired. Compared with whites, blacks had similar odds of having an up-to-date CRC screening. Although not statistically significant, individuals of other races had lower odds of having an up-to-date CRC screening than whites. No difference was noted between men and women.

Logistic regression analysis was performed for each individual screening test (FOBT, sigmoidoscopy, and colonoscopy) to determine whether there were differences in up-to-date screening by BMI. No differences were found for colonoscopy or sigmoidoscopy. For FOBT in the past year, people who were obese had lower odds of being screened (OR, 0.73; 95% CI, 0.59–0.90) (data not shown).

## Discussion

Our results show that 64.9% of Maryland adults aged 50 years and older are up-to-date with CRC screening based on FOBT, sigmoidoscopy, or colonoscopy recommendations for those at average risk. DCBE was not included and may have increased the up-to-date percentage slightly. The majority of Maryland adults aged 50 and older were found to be either overweight (36.0%) or obese (25.4%). The analysis demonstrates that overweight individuals have similar odds of having up-to-date CRC screening as normal and underweight adults. Our analysis also suggests a potential for lower odds of up-to-date screening for obese adults than normal and underweight adults, although this result was not statistically significant. Significant predictors of an up-to-date CRC screening in the multivariable analysis included older age, having some college education or more, and being retired. In addition, we observed that having had a physical examination in the last 2 years and reporting a CRC screening recommendation from a doctor or other health professional were strongly associated with having an up-to-date CRC screening.

Compared with the U.S. population in 2000 (24), adults aged 50 and older living in Maryland have a much higher up-to-date CRC screening rate (34.0% for the U.S. population vs 64.9% for Marylanders). The high screening rates among Marylanders may be due to the high rates of health insurance coverage as well as to high socioeconomic status. Medicare pays for CRC screening, including colonoscopy. In addition, since 2001 Maryland has required health insurance plans to pay for CRC screening; also since 2001, almost all of Maryland's 24 local health jurisdictions — through funding from the Cigarette Restitution Fund Master Settlement Agreement — have funded CRC screening for people with low incomes who are uninsured. CRC incidence rates have shown a steady decline in Maryland since 1997, when the age-adjusted incidence was 61.2 per 100,000, to 2001, when the rate was 52.5 per 100,000. The incidence rate in 2001 was similar to the U.S. rate of 51.8 per 100,000 reported by the National Cancer Institute's Surveillance Epidemiology End Results (SEER). Mortality from CRC also declined in Maryland from 24.5 per 100,000 in 1997 to 21.6 per 100,000 in 2001. The mortality rate in 2001 was statistically significantly higher than the U.S. SEER rate of 20.0 per 100,000. (Rates were age-adjusted to the 2000 standard U.S. population [25].)

In contrast to the results of our study, which show no statistically significant association between BMI levels and CRC screening, previous studies support an association between BMI and cancer screening. Overall, the studies suggest that overweight and obese individuals are more likely to delay or avoid cancer screening (14-17). The authors suggest that there could be multiple factors responsible for this relationship, including concerns about appearance, self-esteem, body image, discomfort with the procedures, and possible negative biases emanating from physicians and health care providers (14). Most of these studies focused on cancer screening among women and demonstrated that overweight and obese women were more likely to delay or avoid Papanicolaou testing, clinical breast examination, and mammography (14-16). Rosen and Schneider (17) examined the relationship between BMI and CRC screening. Their study showed that morbidly obese women (BMI ≥35 kg/m^2^) were less likely to be screened for CRC. Analyzing data from the 1999 BRFSS, Rosen and Schneider used a narrower definition that included FOBT within the last year or endoscopic screening (sigmoidoscopy or colonoscopy) within the last 5 years. The authors also found a low overall CRC screening rate of 43.8% in the United States, whereas our study found a high overall CRC screening rate of 64.9% among Marylanders aged 50 and older.

The high up-to-date CRC screening rate in Maryland could be one reason for our finding of no statistically significant association between BMI levels and an up-to-date CRC screening. We also found a similar distribution of BMIs for those who reported receiving provider screening recommendations and for those who did not report receiving the recommendation, which suggests no demonstrable bias among providers recommending screening for or against people in any particular BMI category. Although a study by Teachman and Brownell (26) found that health professionals may have negative implicit attitudes toward obese individuals, we found no evidence of those attitudes in our analysis based on reported provider recommendations.

Previous studies support some of our significant predictors of an up-to-date CRC screening. In a study by Shapiro et al (22), the authors analyzed data from the BRFSS and found that the reported use of CRC screening tests increased with each decade of age from 50 to 80 and with increasing educational level and income. The study also found that CRC screening rates were not different among whites compared with blacks and that Asian or Pacific Islanders and American Indians or Alaska Natives were less likely to report having a CRC screening test than whites or blacks. Although the odds of having up-to-date CRC screening were lower among people of other races in our study, the result was not statistically significant. It has been shown that self-reported rates of having CRC screening were lower among individuals without health care coverage than among those with health care coverage (22). The study by Zapka et al (23) also determined that individuals who were uninsured had the lowest current CRC screening rate and that the type of insurance had little impact on CRC testing. Our bivariate analysis showed that people with health insurance were almost twice as likely to report up-to-date CRC screening as people without health insurance. However, when recommendation for screening and having a physical examination in the last 2 years were included in the multivariable analysis, having health insurance was not a significant factor. Having had a recent physical examination and, more importantly, having a health care provider recommendation increased the odds of having up-to-date CRC screening. The results of our study, as well as previous studies (23,24), demonstrate that a health care provider recommendation for CRC screening greatly facilitates the screening. Our results also show that about a quarter of all adults aged 50 and older do not report having had CRC screening recommended by a provider, either lower GI endoscopy or a recent FOBT. Therefore, to increase up-to-date CRC screening rates, all physicians should recommend that adults aged 50 and older comply with the guidelines and undergo CRC screening.

Our study has several limitations that could have influenced the results. The MCS data are self-reported by participants, thus introducing the possibility of recall bias. The accuracy of participants' recall of sociodemographic characteristics, height and weight, and provider recommendation of CRC screening and screening occurrence were not verified, which may have affected the results. For example, it is generally known that individuals tend to underreport weight and overreport height, resulting in underestimation of BMI (27,28). Those who were screened may be more likely to report having had a provider recommend CRC screening.  Furthermore, those who participated in the survey could be different from those who did not participate, resulting in selection bias. Both recall bias and selection bias can underestimate or overestimate the true effect. Finally, the MCS obtains data only from noninstitutionalized English-speaking individuals who live in a household residence with landline telephones, thereby limiting the generalizability of the results.

The study also has several strengths, including the large sample size focusing on the Maryland population aged 50 and older. The MCS primarily uses validated questions chosen from national and state surveys. The survey also provides information on multiple potential confounders.

In conclusion, our study showed no statistically significant association between BMI levels and up-to-date CRC screening. However, data suggest a possible association between obesity and lower odds of an up-to-date CRC screening. Furthermore, the analysis showed that obese adults were significantly less likely to be up-to-date with FOBT than normal and underweight individuals. High up-to-date CRC screening rates in the state of Maryland and the seeming lack of bias among providers who recommend CRC screening may have contributed to our finding of no statistically significant association between BMI levels and up-to-date CRC screening. Additionally, high screening rates in the state may reflect the high rate of health insurance coverage among individuals aged 50 and older and the state's requirement that health insurance plans pay for CRC screening. Our results also suggest that to further increase up-to-date CRC screening rates among adults aged 50 and older, health care providers need to recommend appropriate screening procedures for all patients in this age group. Research on the association between body weight and CRC screening is scarce. Our findings in Maryland may not be generalizable to the nation. For these reasons, additional research is needed to verify our findings.

## Figures and Tables

**Table 1:** Sociodemographic Characteristics of Survey Sample of Marylanders Aged 50 Years and Older (n = 3436) Weighted to the Maryland Population, Maryland Cancer Survey 2002

**Characteristic**	**Unweighted No.^a^ (%)**	**Weighted %**	**(95% CI)**
**Geographic area**			
Urban	2289 (66.6)	77.9	NA
Rural	1147 (33.4)	22.1	NA
**Sex**			
Male	1293 (37.6)	44.9	NA
Female	2143 (62.4)	55.1	NA
**Race**			
White	2711 (78.9)	73.5	NA
Black	597 (17.4)	21.5	NA
Other	128 (3.73)	5.00	NA
**Age, y**			
50-64	1933 (56.3)	58.0	NA
65-74	858 (25.0)	24.6	NA
≥75	645 (18.8)	17.4	NA
**Marital status**			
Married or partner in unmarried couple	1941 (56.8)	66.0	64.2-67.7
Divorced or separated	551 (16.1)	13.5	12.2-14.7
Widowed	769 (22.5)	16.6	15.3-17.9
Never married	157 (4.59)	3.9	3.3-4.6
**Education**			
Less than high school	434 (12.7)	13.0	11.7-14.3
High school graduate or GED	1057 (30.9)	29.7	28.0-31.5
College 1-3 years	718 (21.0)	20.3	18.7-21.8
College graduate	642 (18.8)	19.8	18.2-21.4
Advanced degree	565 (16.5)	17.2	15.8-18.7
**Employment**			
Employed for wages	1219 (35.6)	36.6	34.7-38.5
Self-employed	214 (6.3)	6.4	5.4-7.4
Retired	1590 (46.5)	45.2	43.3-47.1
Not employed	396 (11.6)	11.8	10.5-13.0
**Annual household income, $**			
<25,000	733 (21.3)	19.1	17.6-20.6
25,000 to 34,999	386 (11.2)	10.6	9.4-11.7
35,000 to 49,999	478 (13.9)	14.0	12.7-15.3
50,000 to 74,999	427 (12.4)	13.4	12.0-14.7
≥75,000	748 (21.8)	24.0	22.3-25.8
Don't know/not sure or refused	664 (19.3)	19.0	17.5-20.5

CI indicates confidence interval; NA, not applicable.

a Number of responses for each characteristic does not always equal sample size because of missing values.

**Table 2:** Health-Care–related Characteristics of Survey Sample of Marylanders Aged 50 Years and Older (n = 3436), Weighted to the Maryland Population, Maryland Cancer Survey 2002

**Characteristic**	**Unweighted No.^a^ (%)**	**Weighted %(95% CI)**
**Has health insurance**		
Yes	3280 (95.6)	95.8 (95.0-96.6)
No	150 (4.4)	4.2 (3.4-5.0)
**Has had routine checkup in the last year**		
Yes	2929 (86.8)	86.8 (85.5-88.1)
No	447 (13.2)	13.2 (11.9-14.5)
**Has had routine checkup in the last 2 years**		
Yes	3154 (93.4)	93.7 (92.8-94.6)
No	222 (6.6)	6.3 (5.4-7.2)
**Has reported that a health care provider recommended an FOBT in the last year**		
Yes	1370 (40.5)	40.9 (39.0-42.8)
No	2015 (59.5)	59.1 (57.2-61.0)
**Has reported that a health care provider ever recommended having endoscopy**		
Yes	2153 (63.3)	63.5 (61.6-65.4)
No	1250 (36.7)	36.5 (34.6-38.4)
**Has reported recommendation from a health care provider for CRC screening (FOBT in last year or endoscopy ever)**		
Yes	2502 (72.9)	73.2 (71.5-74.9)
No	931 (27.1)	26.8 (25.1-28.5)
**Self-perceived health status**		
Excellent	499 (14.6)	14.8 (13.4-16.2)
Very good	1117 (32.6)	32.3 (30.5-34.2)
Good	1126 (32.9)	33.3 (31.5-35.1)
Fair	530 (15.5)	14.9 (13.5-16.2)
Poor	152 (4.4)	4.6 (3.8-5.5)

FOBT indicates fecal occult blood test; CI, confidence interval; CRC, colorectal cancer.

a Number of responses for each characteristic does not equal sample size because of missing values.

**Table 3:** Unadjusted Associations Between Potential Confounding or Effect-modifying Variables and Colorectal Cancer (CRC) Screening, Maryland Cancer Survey 2002

**Variable**	**Up-to-Date CRC Screening, Weighted %^a^**	***P *Value^b^**
**Total population**	64.9	NA
**Area of residence**		
Urban	65.5	.14
Rural	62.6	
**Sex**		
Male	65.5	.56
Female	64.3	
**Race**		
White	66.1	.03
Black	63.4	
Other	52.6	
**Age, y**		
50-64	59.5	<.001
65-74	73.8	
≥75	70.2	
**Marital status**		
Married or partner in unmarried couple	66.6	.02
Divorced or separated	60.7	
Widowed	64.2	
Never married	55.4	
**Education**		
High school graduate or less	59.6	>.001
Some college or more	68.8	
**Employment**		
Employed for wages	61.2	>.001
Self-employed	50.5	
Retired	72.8	
Not employed	54.7	
**Health insurance**		
Yes	66.1	<.001
No	35.8	
**Health status**		
Excellent, very good, or good	64.8	.80
Fair or poor	65.4	
**Physical examination in last 2 years**		
Yes	67.3	<.001
No	30.5	
**Recommendation from provider for CRC screening**		
Yes	83.5	<.001
No	11.9	

NA indicates not applicable.

a Weighted to the Maryland population.

b Chi-square test to compare proportions; *a priori* level of significance is *P* < .05.

**Table 4:** Unadjusted Associations Between Potential Confounding or Effect-modifying Variables and Body Mass Index (BMI) Levels, Maryland Cancer Survey 2002

**Variable**	**BMI Level**			***P V*alue^a^**

	**<25 kg/m^2^**	**25-29.9 kg/m^2^**	** ≥30 kg/m^2^**	

	**Weighted %**	**Weighted %**	**Weighted %**	
**Total population**	38.7	36.0	25.4	NA
**Area of residence**				
Urban	39.4	35.6	25.0	.23
Rural	36.0	37.2	26.8	
**Sex**				
Male	32.1	42.2	25.7	<.001
Female	44.3	30.6	25.1	
**Race**				
White	42.2	35.8	22.0	<.001
Black	23.9	36.3	39.8	
Other	49.6	36.7	13.7	
**Age, y**				
50-64	35.1	36.5	28.5	<.001
65-74	39.3	36.4	24.2	
≥75	49.2	33.7	17.0	
**Marital status**				
Married or partner in unmarried couple	37.8	37.9	24.3	.01
Divorced or separated	35.4	35.4	29.2	
Widowed	43.1	30.9	26.1	
Never married	43.9	27.2	29.0	
**Education**				
High school graduate or less	35.9	34.2	29.9	<.001
Some college or more	40.7	37.4	21.9	
**Employment**				
Employed for wages	37.2	36.2	26.6	.43
Self-employed	34.7	41.6	23.8	
Retired	40.4	35.6	24.0	
Not employed	38.5	33.4	28.1	
**Health insurance**				
Yes	39.0	35.9	25.1	.15
No	30.0	38.8	31.2	
**Health status**				
Excellent, very good, or good	39.8	37.0	23.2	<.001
Fair or poor	33.6	31.9	34.5	
**Physical examination in last 2 years**				
Yes	38.5	35.7	25.9	.19
No	42.6	38.1	19.4	
**Recommendation from provider for colorectal cancer screening**				
Yes	38.0	36.1	26.0	.40
No	40.6	35.7	23.7	

NA indicates not applicable.

a Chi-square test to compare proportions; a priori level of significance is P <.05.

**Table 5:** Unadjusted Associations Between Body Mass Index (BMI) Levels and Colorectal Cancer (CRC) Screening, Maryland Cancer Survey 2002

**BMI Category (kg/m^2^)**	**Up-to-Date CRC Screening, Weighted %**	***P *Value^a^**	**Unadjusted Odds Ratio for CRC Screening (95% CI)**
<25 (normal and underweight)	65.4	.84	Ref
>25-29.9 (overweight)	65.3		1.03 (0.87-1.21)
≥30 (obese)	64.0		0.92 (0.76-1.10)

CI indicates confidence interval; ref, reference group.

a Chi-square test for comparison of screening prevalence across different BMI categories.

**Table 6:** Associations Between Body Mass Index (BMI) Levels and Other Factors and Up-to-Date Colorectal Cancer (CRC) Screening in Two Multivariable Logistic Regression Models, Maryland Cancer Survey 2002

**Category**	**Model 1^a^**		**Model 2^b^**	

	**OR (95% CI)**	***P *Value**	**OR (95% CI)**	***P *Value**
**BMI (kg/m^2^)**				
<25 (normal and underweight)	Ref		Ref	
25-29.9 (overweight)	1.07 (0.89-1.27)	.48	1.05 (0.83-1.33)	.70
≥30 (obese)	1.07 (0.88-1.30)	.52	0.84 (0.65-1.09)	.19
**Sex**				
Female	Ref		Ref	
Male	0.94 (0.80-1.11)	.49	1.01 (0.81-1.25)	.96
**Race**				
White	Ref		Ref	
Black	0.98 (0.80-1.21)	.88	1.16 (0.87-1.55)	.31
Other	0.73 (0.49-1.08)	.11	0.77 (0.46-1.29)	.33
**Age, y**				
50-64	Ref		Ref	
≥65	1.43 (1.17-1.74)	<.001	1.45 (1.10-1.90)	.008
**Marital status**				
Not married	Ref		Ref	
Married	1.43 (1.22-1.67)	<.001	1.16 (0.94-1.44)	.17
**Education**				
High school graduate or less	Ref		Ref	
Some college or more	1.62 (1.38-1.90)	<.001	1.32 (1.07-1.64)	.01
**Employment**				
Retired	Ref		Ref	
Employed for wages	0.67 (0.55-0.83)	<.001	0.63 (0.47-0.83)	.001
Self-employed	0.46 (0.33-0.64)	<.001	0.49 (0.32-0.76)	.002
Not employed	0.59 (0.45-0.77)	<.001	0.68 (0.48-0.98)	.04
**Geographic area**				
Rural	Ref		Ref	
Urban	1.26 (1.07-1.48)	.006	1.23 (0.99-1.53)	.06
**Health insurance**				
Yes	—		0.98 (0.58-1.68)	.95
No	—		Ref	
**Physical examination in last 2 years**				
Yes	—		2.53 (1.65-3.90)	<.001
No	—		Ref	
**Screening recommendation from provider**				
Yes	—		36.7 (28.7-47.0)	<.001
No	—		Ref	

OR indicates odds ratio; CI, confidence interval; ref, reference group; dash (—), not included in model.

a Model 1 includes only sociodemographic characteristics.

b Model 2 includes sociodemographic characteristics and health-care–related variables.
