# Erratum, Vol. 3: No. 3

**Published:** 2006-12-15

The name of Annette Hopkins was omitted from the author affiliations list for the article "Body Mass Index and Up-to-Date Colorectal Cancer Screening Among Marylanders Aged 50 Years and Older." The correct author affiliations list appears below:

Author Affiliations: Mikhail Menis, Bernard Kozlovsky, Pat Langenberg, Min Zhan, Ebenezer Israel, Annette Hopkins, Department of Epidemiology and Preventive Medicine, University of Maryland School of Medicine, Baltimore, Md; Diane M. Dwyer, Carmela Groves, Center for Cancer Surveillance and Control, Maryland Department of Health and Mental Hygiene, Baltimore, Md.

The author affiliations list was corrected on our Web site on October 13, 2006, and appears online at http://www.cdc.gov/pcd/issues/2006/jul/05_0178.htm. We regret any confusion this error may have caused.

